# Farewell to Wigan Matron

**Published:** 1921-02-12

**Authors:** 


### Farewell to Wigan Matron.

THE farewell to Miss Coggins, the matron of the Royal Albert Edward Infirmary, Wigan, on her departure to take up the matronship of the West London Hospital, took place on January 31. The Chairman and members of the Board of Management, together with the medical and nursing staffs, made her a presentation through the Chairman, who voiced the regret of all at the loss of Miss Coggins' valuable services, and particularly referred to the excellent work she had done in trying circumstances during the war. In the evening Miss Coggins gave a dance to the nursing staff and friends in the Nurses' Home, which was taken part in with much zest and thoroughly enjoyed.

